# Chronic shifts in the length and phase of the light cycle increase intermittent alcohol drinking in C57BL/6J mice

**DOI:** 10.3389/fnbeh.2015.00009

**Published:** 2015-02-03

**Authors:** Joshua J. Gamsby, Danielle Gulick

**Affiliations:** ^1^Byrd Alzheimer’s Institute, University of South Florida HealthTampa, FL, USA; ^2^Department of Molecular Medicine, Morsani College of Medicine, University of South FloridaTampa, FL, USA

**Keywords:** alcoholism, circadian rhythms, mouse model, addiction, environmental desynchrony

## Abstract

**Introduction**: Shift workers—e.g., health care professionals, truck drivers, and factory workers—are forced to maintain daily cycles at odds with their natural circadian rhythms and as a consequence need to frequently readjust these cycles. This shift work-induced circadian desynchrony (CD) is associated with increased sleep disorders and with alcohol abuse. Nonetheless, it has proven difficult to model CD-induced changes in alcohol consumption in mouse models, which is an important step toward identifying the mechanisms by which CD increases alcohol intake. This study examined whether frequent changes in the light cycle could increase free access alcohol intake in a mouse line that readily consumes alcohol.

**Methods**: Free access alcohol intake, water intake, and wheel-running activity patterns of male C57BL/6J mice were measured while the mice were maintained on a normal 12HR photoperiod for baseline data for 2 weeks. The mice were then exposed to an alternating photoperiod of 12 h and 18 h, with light onset advanced 8 h during the 18HR photoperiod. The photoperiods rotated every 3 days, for 21 days total.

**Results**: The repeated pattern of phase advances and delays, with a concurrent change in the length of the photoperiod, shifted mice to a pattern of intermittent alcohol drinking without altering water intake. Wheel running activity demonstrated that mice were unable to reset their behavioral clocks during CD, showing constant, low-level activity with no peak in activity at the start of the dark phase and greater activity during the morning light phase.

**Conclusion**: It is possible to model CD effects on alcohol intake in C57BL/6J mice using a pattern of phase shifts and changes in the photoperiod. Using this model, we demonstrate that mice begin intermittent drinking during CD, and this increase in alcohol intake does not correlate with an increase in overall activity or in overall fluid intake.

## Introduction

Despite the well-recognized and devastating effects on health and quality of life, alcohol use disorder (AUD) remains a major societal problem and financial burden in the United States, with alcohol abuse-related costs estimated to be $224 billion annually (Bouchery et al., 2011). AUD has proven difficult to treat because of its complex etiology, with attributions to a variety of genes and environmental factors. One such factor that has been implicated in the etiology of alcoholism is the disruption of circadian rhythms, whether at the genetic or environmental level (Crum et al., 2004a,b; Shibley et al., 2008; Chokroverty, 2010). Circadian rhythms evolved as a way to cope with our planet’s 24 h axial rotation and function to keep internal time synchronized with the external environment. Although circadian rhythms can be reset by external cues called zeitgebers, such as sunlight, they will continue to oscillate with a period of approximately 24 h in the absence of these cues. In mammals, the master circadian clock resides in the suprachiasmatic nucleus (SCN) of the anterior hypothalamus and governs the timing of many homeostatic functions such as metabolism, body temperature, and the sleep/wake cycle. Disruptions in these functions are associated with a wide variety of physical, mental, and emotional disorders, including substance abuse and dependence (Falcón and McClung, 2009). Specifically, there are direct correlations of both sleep disruptions and changes in circadian gene expression with increases in alcohol drinking behaviors and increased sensitivity to alcohol (Benca et al., 1992; Wirz-Justice et al., 2001; Spanagel et al., 2005; Falcón and McClung, 2009; Perreau-Lenz et al., 2009; Agapito et al., 2010; Kovanen et al., 2010).

Clinically, 36–72% of patients presenting with AUD also suffer from insomnia and sleep disruption (Brower, 2003), but there is a question of causality to the connection between circadian desynchrony (CD) and the development of AUD. For example, people under intense stress may start drinking to relax, but alcohol worsens sleep quality and negatively impacts physical and emotional health, decreasing resilience to the stress that led them to drink in the first place. Chronic alcohol abuse can also disrupt circadian rhythms and aggravate underlying sleep disorders (Costa e Silva et al., 1996) because alcohol disrupts sleep cycles, eating habits, and homeostatic rhythms, such as those in body temperature and metabolic function (Rosenwasser, 2001; Wasielewski and Holloway, 2001; Zhou et al., 2014). In other cases, the sleep disturbance precedes alcohol abuse, because patients with sleep disorders first begin to abuse alcohol as a sleep aid (Johnson et al., 1998). Regardless of the precipitating event, CD and AUD form a self-perpetuating cycle of sleep disruption and alcohol abuse. Aberrant daily schedules, such as those required by shift work, are also implicated in the risk of AUD (Morikawa et al., 2013). Clinically, the International Classification of Sleep Disorders: Diagnostic and Coding Manual (ICSD-2) describes alcohol and drug abuse as disorders that often occur concomitantly with both jet lag and shift work (American Academy of Sleep Medicine, 2005).

Not surprisingly, the mental and physical stress of shift work leads individuals to drink both to relax and as a sleep aid (Harrington, 1994). Indeed, Blomeyer et al. recently demonstrated that single nucleotide polymorphisms in a key circadian gene, *Period2*, only predict increased alcohol intake in individuals under high stress, suggesting an essential role of environment in mediating the effects of circadian genotype on behavior (Blomeyer et al., 2013). People who do shift work are forced to maintain daily cycles at odds with their natural circadian rhythms and some must frequently readjust these cycles. Shift work-induced CD is associated with increased sleep disorders, and in some cases, with heavy alcohol abuse (Morikawa et al., 2013).

Although the clinical correlation between CD and alcohol abuse seems concrete, this connection has proven difficult to recapitulate in animals models. As previously mentioned, one of the defining principles of a circadian rhythm is that it can be reset or “entrained” by external environmental cues, such as light or temperature exposure. Changes in these cues affect alcohol intake in rodent models, but the variability in experimental design—species/strain, protocol—makes it difficult to collectively interpret the current literature. For example, repeated phase advances (e.g., mimicking eastbound jet lag) failed to alter alcohol intake in mice (Rosenwasser and Fixaris, 2013). However, long-term testing in Sprague-Dawley rats revealed increases in alcohol intake following both phase advances and phase delays, as well as following rotating photoperiods (Gauvin et al., 1997). The latter studies used paradigms more closely mimicking the CD experienced in everyday situations such as shift work and travel, and support the clinical evidence that shift work increases susceptibility to addiction. Based on previous work, and the review of methods to study circadian rhythms in rodents by Jud et al. (2005), the current study assesses changes in alcohol drinking in the alcohol-preferring C57BL/6J mouse during a period of repeated phase advances and delays with concurrent changes in the photoperiod.

## Material and methods

### Subjects

Male C57BL/6J mice (Jackson Laboratory, Bar Harbor, ME) were tested at 8–13 weeks of age (20–30 g). Mice were housed individually for 2 weeks prior to the start of the study with* ad libitum* access to food and water. A 12-h light:dark cycle (lights on at 19:00) was maintained from this initial habituation through the baseline measurement period. There was a 1-week habituation to the light cycle, after which mice were given access to a second bottle containing 2% alcohol (v/v). The alcohol concentration was doubled to 4% after 4 days and then doubled again to 8% after 4 more days. After 1 week of drinking 8% alcohol, mice were individually housed in dual lickometer phenotyping chambers (Lafayette Instruments, Lafayette, IN) that measured wheel running, alcohol intake, and water intake. Mice had free access to the running wheel, food, alcohol, and water throughout the study. The cages were housed in sound-attenuating plant growth incubators (Thermo Scientific, Waltham, MA) that allowed precise control of the luminance and temperature on an hourly and daily basis. All experiments were carried out in accordance with the National Institutes of Health guide for the care and use of Laboratory animals (NIH Publications No. 8023, revised 1978) and were approved by the University of South Florida Institutional Animal Care and Use Committee.

### Experimental design

Mice were placed into the phenotyping cages on a reversed (19:00–7:00) photoperiod for 12 days in order to assess baseline behavioral patterns. On the 13th day, the dark phase was shortened and advanced (15:00–21:00; 18HR light). After 3 days, the original light phase (19:00–7:00; 12HR light) was restored for 4 days. This sequence of a phase advance followed by a phase delay and concurrent shift in period was repeated for 21 days.

### Data acquisition and analysis

Data from the lickometers and the running wheel were recorded in 5 min bins by a computer running Activity Wheel Monitor Software (Lafayette Instruments, Lafayette, IN). Health checks occurred with the use of infrared goggles during the dark phase of each cycle. At the same time as the health check, the data was downloaded from the computer for later analysis and the alcohol bottles were weighed to measure the volume of alcohol intake. At the end of the experiment, all data were compiled and analyzed using the MATLAB (MathWorks, Natick, MA) software extension CLOCKLAB (Coulbourn Instruments, Whitehall, PA). CLOCKLAB actograms and light/temperature data from HOBO Pendant Temperature/Light Data Loggers (Onset, Cape Cod, MA) were examined to ensure no unexpected spikes in light, temperature, or behavior occurred during the study.

Alcohol intake (g/kg/min in bouts) was calculated using the volume consumed over 24 h (calculated by recording the volume in each bottle at the time of the health check—approximately 2 µL per count), the body weight at the start of the experiment, and the counts per min in bouts. Onsets of activity were analyzed by 1-way analysis of variance (ANOVA). All data points were binned by hour or by bout and the following variables were analyzed by repeated measures analysis of variance (rmANOVA): counts per hour, bout length, counts per bout, bouts per day, count rate in bouts, alcohol g/kg intake in bouts, and total counts per day. In order to delineate the bouts, they were defined as the period during which there were a minimum 5 counts/min and a maximum gap of 5 min between behaviors. Tukey’s *post hoc* analysis was used to detect significant differences at *p* < 0.05 (SPSS version 22; Chicago, IL).

## Results

### Overall activity

**Figure 1:**
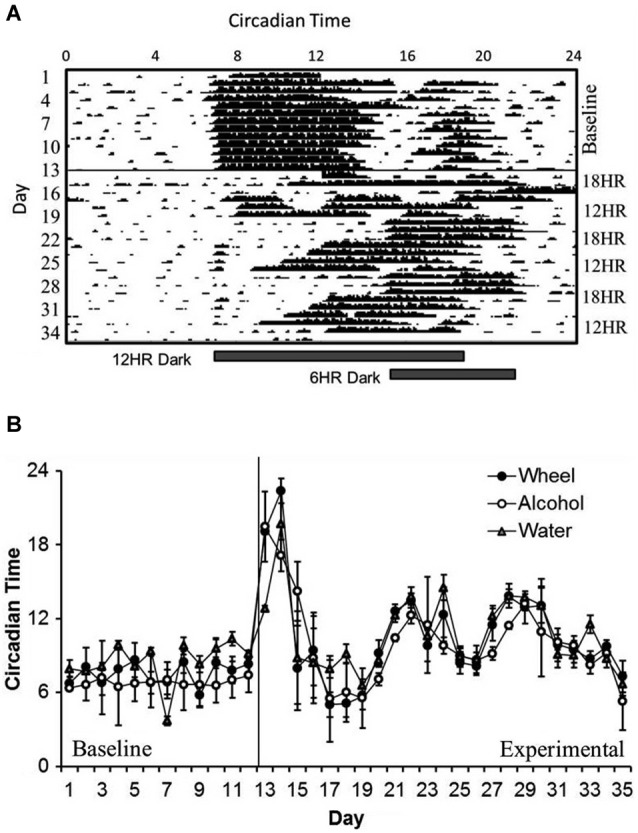
**Circadian patterns and period onsets in C57BL/6J mice during baseline alcohol access and environmental circadian desynchrony (CD). (A)** Representative actogram of wheel-running data from 1 mouse. Activity period remained stable throughout the baseline period, with activity onsets at approximately the same time that lights were turned off (7:00). During CD, mice were constantly shifting their behavior onsets to try to compensate for the phase advances and delays between the 12HR and 18HR photoperiods. **(B)** Over the course of the experiment, onset times for all three behaviors (wheel-running, alcohol intake, and water intake) remained synchronized, although these onsets varied dramatically during the experimental photoperiods (data indicate the mean ± standard error of the mean (SEM) for *n* = 8).

In examining overall patterns of activity across the length of the experiment, there were no significant differences in the period length or the time of activity onset between wheel-running, alcohol intake, or water intake. During the baseline photoperiod, mice showed normal circadian rhythms in all three measures (mean period: 23.91, SD: 0.077). During the CD photoperiods, there was a small but significant increase in the length of the period (mean period: 24.04, SD: 0.038; different from baseline at *p* < 0.05). As expected, mice showed no significant daily variations in the time of activity onset during baseline (see Figure 1A for a representative wheel-running actogram), but the phase of onsets oscillated dramatically during the CD photoperiods (*F*_(3,32)_ = 8.27, *p* < 0.001; Figure 1B). The greatest shift in onset was during the first 18HR light cycle; after this, mice never completely reset their activity patterns to match the shifting light cycles, with onsets vacillating as early as 6:00 and as late as 15:00. It is important to note that there are no non-alcohol drinking controls in this study, so the changes in circadian rhythms may be impacted by chronic exposure to alcohol as well as by CD.

### Activity patterns

Unlike the period lengths and the phase of activity onsets, which were nearly identical across measures, we observed contrasting patterns of wheel-running, alcohol intake, and water intake behaviors over the circadian cycle.

**Figure 2:**
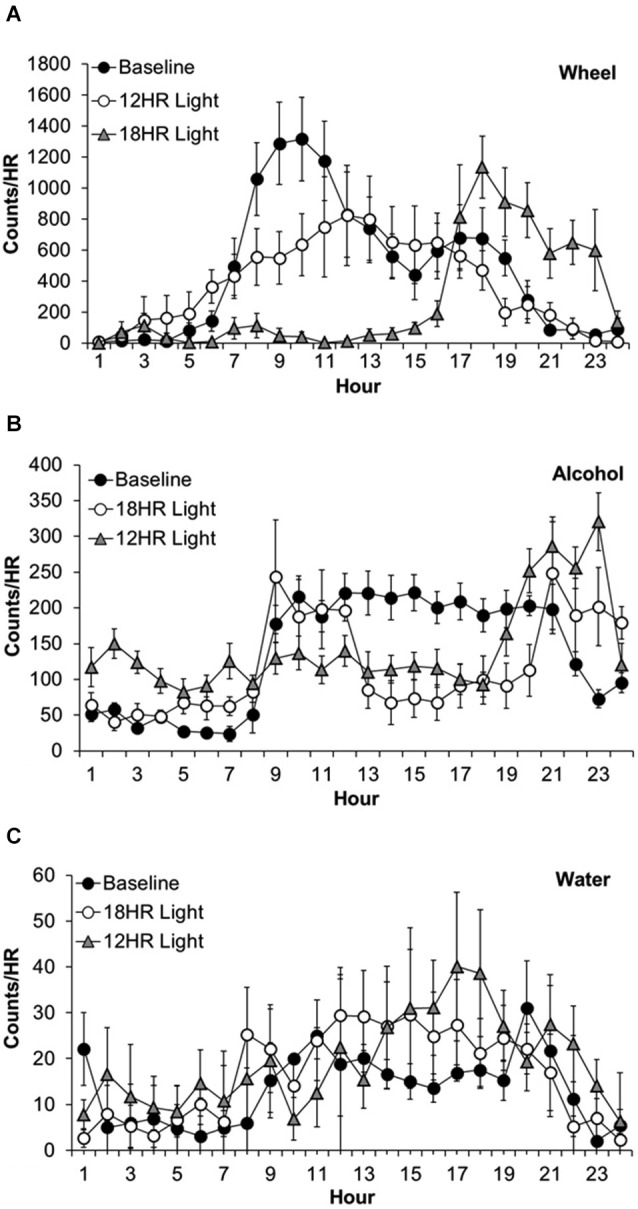
**Overall activity counts by hour in C57BL/6J mice during baseline alcohol access and environmental CD. (A)** Patterns of wheel-running behavior varied dramatically between the baseline and experimental photoperiods, with lower activity in the 18HR photoperiod compared to baseline from 7:00–13:00, and greater activity from 17:00–23:00. Mice also showed lower activity in the 12HR photoperiod compared to baseline from 8:00–10:00. **(B)** Patterns of alcohol drinking also varied by photoperiod. From 24:00–7:00, mice displayed greatest activity in the 18HR photoperiod. Mice were also more active in the 12HR photoperiod compared to baseline from 5:00–7:00. Alcohol intake in the 18HR photoperiod was significantly lower than baseline from 11:00–20:00, and in the 12HR photoperiod it was significantly lower than baseline from 12:00–20:00. From 21:00–24:00, drinking during both CD photoperiods was higher than at baseline. **(C)** There were few changes in water intake between photoperiods. The only significant difference was higher water intake at 18:00 in the 12HR photoperiod compared to baseline (data indicate the mean ± SEM for *n* = 8).

Wheel-running activity varied by hour, *F*_(23,463)_ = 12.63, *p* < 0.001 and the activity by hour varied across photoperiods, *F*_(46,437)_ = 9.78, *p* < 0.001, although there was no overall difference in wheel-running between photoperiods. Mice showed lower activity in the 18HR photoperiod compared to baseline from 7:00–13:00, and greater activity from 17:00–23:00 (*p* < 0.001; Figure 2A). Mice also showed lower activity in the 12HR photoperiod compared to baseline from 8:00–10:00 (*p* < 0.001). Overall, mice showed an attenuated oscillation in wheel-running in the 12HR photoperiod, and more robust activity peaks during the baseline and 18HR photoperiods.

Alcohol drinking varied by hour, *F*_(23,463)_ = 4.84, *p* < 0.001 and the alcohol intake by hour varied across experiment photoperiods, *F*_(46,437)_ = 14.56, *p* < 0.001, although there was no overall difference in alcohol intake between photoperiods (Figure 2A). During the first 7 h of the day, the mice displayed greatest alcohol drinking in the 18HR photoperiod (*p* < 0.001; Figure 2B) and greater alcohol drinking in the 12HR photoperiod compared to baseline from 5:00–7:00 (*p* < 0.001). There were no differences from 8:00–10:00. Alcohol intake in the 18HR photoperiod was significantly lower than baseline from 11:00–20:00, and in the 12HR photoperiod it was significantly lower than baseline from 12:00–20:00 (*p* < 0.001). From 21:00–24:00, drinking during both CD photoperiods was higher than at baseline (*p* < 0.001). Overall, mice showed a lower amplitude oscillation in alcohol drinking during the CD photoperiods, with greater alcohol intake during the light phase.

Water drinking varied by hour, *F*_(23,463)_ = 2.36, *p* < 0.001 and the water intake by hour varied across experiment photoperiods, *F*_(46,437)_ = 2.80, *p* < 0.001, although there was no overall difference in water intake between photoperiods. However, the only significant difference was higher water intake in 18:00 in the 12HR photoperiod compared to baseline (*p* < 0.05; Figure 2C). Overall, mice showed low levels of water drinking behavior compared to wheel-running and alcohol drinking, with no major differences in activity across the baseline and CD photoperiods.

### Bout analysis

While overall activity patterns (measured as the average wheel counts per hour) can help us assess the global effects of CD on behavior, one key aspect of modeling alcohol abuse is the ability to demonstrate changes in the specific pattern of alcohol intake. Thus, we next performed a bout analysis to determine whether behaviors were occurring in short, intense periods (bouts) or in longer, more conservative periods.

**Figure 3:**
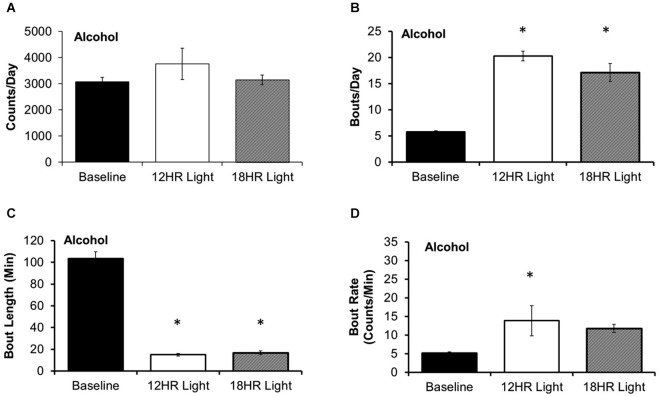
**Bout analysis of alcohol drinking by photoperiod. (A)** There were no differences in the total counts on the alcohol lickometer between photoperiods. **(B)** Mice increased the number of bouts per day during both CD photoperiods. **(C)** Balancing the increased number of bouts, mice drank alcohol in shorter bouts in the CD photoperiods. **(D)** The rate of licks in each bout only increased in the 12HR photoperiod (data indicate the mean ± SEM for *n* = 8; asterisks indicate significant differences from baseline at *p* = 0.05).

There was no significant effect of photoperiod on the total counts per day on the alcohol bottle (Figure 3A). There were significant effects of photoperiod on the number of bouts per day (*F*_(2,19)_ = 53.85, *p* < 0.001; Figure 3B), the bout length (*F*_(2,19)_ = 203.72, *p* < 0.001; Figure 3C), and on the bout rate, the number of counts/min in each bout (*F*_(2,19)_ = 3.99, *p* < 0.05; Figure 3D), and the alcohol intake (g/kg) per minute in each bout (*F*_(2,19)_ = 14.21, *p* < 0.001; Table 1). Mice drank alcohol in shorter bouts during both experimental photoperiods (*p* < 0.001) compared to baseline, but they compensated by increasing the number of bouts in both photoperiods (*p* < 0.001). This suggests that mice transitioned to a pattern of intermittent drinking during CD. Although the actual rate of licking in these bouts only increased significantly in the 12HR photoperiod (*p* < 0.05), there was a trend towards a faster lick rate in the 18HR photoperiod (*p* = 0.1), and mice drank significantly more alcohol per min in bouts during both the 12HR and 18HR photoperiods compared to the baseline period (*p* < 0.05). There were no significant differences in alcohol intake (g/kg) over the 24 h day (Table 1). While we were unable to assess blood alcohol levels without disturbing the circadian rhythms of the mice, the volume of alcohol consumed in the bouts is comparable to the volumes consumed in other studies of limited access binge drinking in mice, as discussed below.

**Table 1:** **Analysis of alcohol intake in bouts (g/kg/min and total g/kg) and over 24 h (g/kg), based on photoperiod: Mice drank at a significantly higher rate, but drank less total alcohol per bout, during both the 12HR and 18HR photoperiods compared to the baseline period**.

Photoperiod	Alcohol intake g/kg/min in bouts	SEM	Alcohol intake g/kg in bouts	SEM	Alcohol intake g/kg/day	SEM
Baseline	0.030	0.001	3.099	0.029	17.68	0.25
12HR Light	0.065*	0.008	0.985*	0.065	21.60	1.33
18HR Light	0.061*	0.003	1.037*	0.061	18.04	0.47

There was no difference in total alcohol intake over the 24-h day (data indicate the mean and the standard error of the mean (SEM) for n = 8); * indicates a significant difference from baseline.

**Figure 4:**
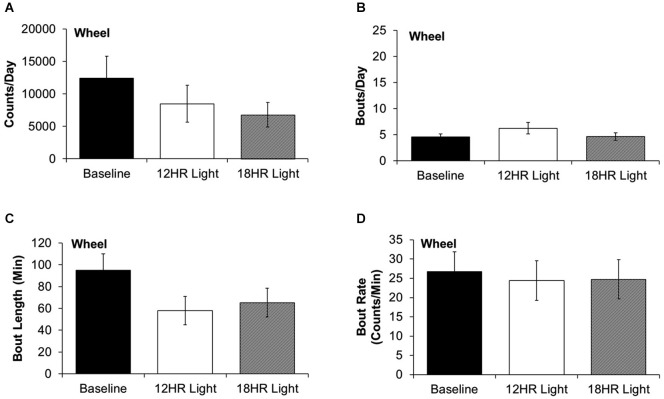
**Bout analysis of wheel-running by photoperiod. (A)** There were no differences in the total counts on the wheel counter between photoperiods. **(B)** There were no differences in the number of bouts per day. **(C)** There were no differences in the bout lengths. **(D)** There were no differences in the rate of wheel rotations in bouts (data indicate the mean ± SEM for *n* = 8).

There were no significant differences between photoperiods in the bout analysis of wheel running. There were non-significant trends towards fewer counts per day in the 18HR photoperiod (*F*_(2,19)_ = 1.52, *p* = 0.2; Figure 4A) and shorter bout lengths (*F*_(2,19)_ = 2.28, *p* = 0.3; Figure 4C) during both CD photoperiods. There was no change in the number of bouts per day (Figure 4B) or in the bout rate (Figure 4D). Thus, the change in alcohol drinking behavior does not reflect an overall change in activity patterns, as there were asymmetrical changes in wheel-running and alcohol drinking behaviors.

**Figure 5:**
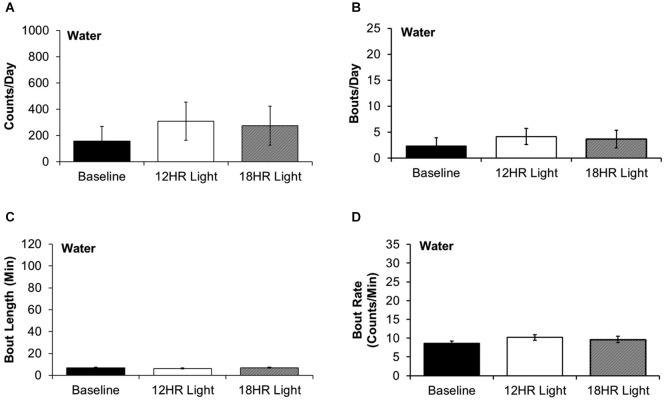
**Bout analysis of water drinking by photoperiod. (A)** There were no differences in the total counts on the water lickometer between photoperiods. **(B)** There were no differences in the number of bouts per day. **(C)** There were no differences in the bout lengths. **(D)** There were no differences in the rate of licks in bouts (data indicate the mean ± SEM for *n* = 8).

There were no significant differences between photoperiods in the bout analysis of water drinking (Figures 5A–D). This suggests that mice selectively switch to intermittent alcohol drinking during CD.

## Discussion

This study demonstrates that CD is sufficient to increase binge-like alcohol intake in C576BL/6J mice. This change in alcohol drinking occurs independently of any change in wheel-running or water drinking behavior, demonstrating a selective increase in alcohol seeking. The development of this model of CD-induced intermittent drinking provides a foundation to assess how changes in circadian cycles, whether they result from jet lag, work shifts, or academic schedules, may lead to alcohol abuse and dependence. This model may prove useful to future studies assessing the mechanistic connections between CD and alcoholism or testing potential therapies to target CD in the treatment of alcoholism.

Previous studies have focused primarily on animal models of genetic circadian disruption, which demonstrate innate tendencies toward high alcohol intake. For example, polymorphisms in the circadian *Period* genes increase alcohol intake in mice bred on an alcohol-preferring C57BL/6J background (Wasielewski and Holloway, 2001; Crum et al., 2004a; Spanagel et al., 2005; Zghoul et al., 2007; Dong et al., 2011; Wang et al., 2012; Gamsby et al., 2013). Similarly, men diagnosed with severe alcohol dependence show lower overall expression of circadian genes than nonalcoholic controls (Spanagel et al., 2005). In a large-scale single nucleotide polymorphism study, Kovanen et al. (2010) demonstrated significant associations between polymorphisms in the circadian gene *BMAL1* and social drinking, and between polymorphisms in *Period2* and alcohol dependence. Thus, the neuroadaptive processes that lead to addiction (Hyman et al., 2006; Falcón and McClung, 2009) may result, in part, from changes in circadian function. The development of models of CD are important to this research field because shift work- and jet lag-based circadian rhythm disorders are by far the most common, compared to genetic circadian rhythm disorders (Barion and Zee, 2007). Taken in combination with the existing literature on CD and alcohol abuse, the current study suggests that CD-induced increases in alcohol intake occur regardless of whether the circadian disruption is genetic or environmental.

Other studies have used different protocols to model CD and alcohol intake, with disparate results. For example, a repeated sequence of 8 6-h phase advances (e.g., mimicking eastbound jet lag once weekly) failed to alter intake of 10% alcohol in C57BL/6J mice (Rosenwasser and Fixaris, 2013). However, these mice never showed an alcohol preference (maximum preference vs. water ~45% in the Rosenwasser study, as opposed to ~92% in the current study). This low alcohol preference suggests that alcohol was not rewarding nor anxiolytic in this protocol, and thus it is not surprising that mice did not increase their alcohol intake during CD. In a separate set of experiments, long term testing in Sprague-Dawley rats revealed increases in alcohol intake following both phase advances and phase delays of the light cycle, as well as following rotating light cycles (such as those experienced by shift workers, Gauvin et al., 1997). Although alcohol consumption is lower in Sprague-Dawley rats than in many mouse lines, this study demonstrated the capacity for changes in alcohol intake during a sequence of phase advances and phase delays.

The current study demonstrates face validity for CD-induced increases in alcohol intake, and more work is needed to identify the mechanisms by which this occurs. Many circadian researchers hypothesize that the mental and physical stress of shift work has a causal role in alcohol abuse, as individuals drink both to relax and as a sleep aid (Harrington, 1994; Johnson et al., 1998). Blomeyer et al. recently demonstrated that the polymorphisms in the *Period2* gene only predict increased alcohol intake in individuals under high stress, suggesting that stress is certainly a factor in alcohol abuse. To assess the role of stress in our model of CD-induced alcohol intake, future work will assess how our CD protocol alters plus-maze anxiety behavior and forced swim helplessness behavior. Nonetheless, other factors may also play a role in this relationship. For example, Gauvin et al. (1997) demonstrated changes in blood alcohol metabolism across the circadian day independent of food intake patterns. Although we saw a shift to intermittent alcohol drinking independent of overall activity in the current study, we did not assess blood alcohol concentrations, as such intervention would have reset the circadian rhythms of the mice. Thus, we are only able to report behavioral observations. However, we found that mice drank physiologically relevant volumes of alcohol during the experimental photoperiod bouts, as high as 0.06 g/kg/min. In Grahame and Grose (2003), mice drank 0.5 g/kg/min on average during 30 min intermittent access to alcohol. Similarly, in Becker and Lopez (2004), mice drank 0.35 g/kg/min on average during 30 min intermittent access to alcohol. While these studies only provide a broad comparison the current research, it does suggest that mice exposed to CD drink at levels comparable to mice in limited access alcohol paradigms. Future studies will assess blood alcohol concentrations in mice at the terminal point in a similar study.

This study demonstrates a protocol to induce robust patterns of intermittent alcohol drinking in an alcohol-preferring C57BL/6J mouse line. Although alcohol is available *ad lib* to the mice, and such free access is not typically considered a model of alcohol abuse, the mice self-select binge drinking behaviors, which is arguably a more valid model of human binge drinking, as this binge drinking is most commonly the result of individual choice rather than limited access to alcohol. Future studies will assess the mechanisms underlying the environmental CD-induced increase in alcohol drinking, in order to identify novel targets to reduce alcohol consumption in the proportion of the population challenged by CD.

## Conflict of interest statement

The authors declare that the research was conducted in the absence of any commercial or financial relationships that could be construed as a potential conflict of interest.
